# The usefulness of the basophil activation test for monitoring the effectiveness of wasp venom immunotherapy in different age groups

**DOI:** 10.1002/clt2.70073

**Published:** 2025-08-13

**Authors:** Andrzej Bozek, Martyna Miodonska, Aleksandra Mitka, Dominika Sadowska, Janne Winterstein, Radosław Gawlik, Marita Nittner‐Marszalska

**Affiliations:** ^1^ Clinical Department of Internal Diseases, Dermatology and Allergology Medical University of Silesia Katowice Poland; ^2^ Allergy Outpatient Clinic, Research Department Munich Germany; ^3^ Clinical Department of Internal Disease, Allergology and Immunology Medical University of Silesia Katowice Poland; ^4^ Department of Internal Medicine, Pneumology and Allergology Wroclaw Medical University Wrocław Poland

To the Editor,

Allergen immunotherapy (AIT) remains a well‐established and widely used approach for treating immediate type allergies including allergic rhinitis and conjunctivitis, certain forms of allergic asthma, select food allergies, and allergic reactions to Hymenoptera insect venom immunotherapy (VIT). Currently, VIT is the only causative and, in some cases, can be life‐saving.^1^

AIT has been proven to be effective for most age groups, including patients over 60 years old.^2^ However, limited studies have confirmed the safety and effectiveness of VIT in these populations.^2^ This is particularly important in cases of anaphylactic reactions following an insect sting, which is an indication for VIT also in the oldest patients.^3^

Therefore, evaluating of the effectiveness of VIT and the rate at which tolerance to the venom develops is of key importance. The basophil activation test (BAT) can be the optimal tool for such an evaluation. The BAT is a valuable tool for final qualification for VIT and often resolves doubts.^4^, ^5^ The most diagnostically valuable method for assessing the effectiveness of VIT is the live insect sting challenge (SP). However, SP has significant limitations, including the necessity of conducting it in highly specialized centers, the risk of complications, including those related to the application of the full, unfractionated dose of the allergen, and the potential risk of reactivating the allergic state. Unlike SP, the BAT for assessing VIT effectiveness is free of these limitations.^6^

The authors would like to present an assessment of the efficacy of VIT for wasp venom after 1 year of treatment in different age groups, comparing the efficacy in young and old patients to identify potential differences. This builds of an earlier observational study, which confirmed comparable efficacy after 6 months of VIT initiation in people aged 18–35 and over 60 years in the BAT test.^7^ These results were independent of the type of vaccine used Venomenhal (Hal Allergy) or Diater.

In the second part of observation, the study group was slightly reduced (drop‐out due to patients' resignation despite the effectiveness of the treatment and the lack of adverse effects), and its final characteristics are presented in Table 1.

**TABLE 1:** Characteristic of the study population after 12 months of VIT (induction and maintenance): older (over 60 years) versus young (18–35 years).

Parameters	Older patients	Young patients	*p*
	*n* = 53	*n* = 51	
Median age (range)	67 (61–76)	27 (18–35)	p* = 0.01
Female, *n* (%)	19 (36)	20 (39)	*p* = 0.45
Number of insect sting systemic reactions acc. Muller (%) prior VIT			
I	3.1	2.8	*p* = 0.55
II	25.1^a^	18.8	*p* = 0.04
III	40.3	39.8	*p* = 0.72
IV	31.5	38.6^b^	*p* = 0.02
Average number of injections during VIT	21	21	*p* = 0.47
% of patients using beta antagonists	22	5^c^	*p* = 0.01
% of patients using inhibitors of ACI	29	4^c^	*p* = 0.01
Median total IgE serum concentration kU/L (range)	68.5 (5.1–3210.0)	72.1 (23.1–977.0)	p* = 0.05
Median IgE against wasp venom extract concentration kU/L (range)	16.34 ± 11.2 (0.0–100.0)	15.3 ± 6.6 (0.8–100.0)	p* = 0.21
Median rVes v1 concentration kU/L (range)	8.5 (0.9–100.0)	9.1 (0.0–99.1)	p* = 0.06
Median rVes v5 concentration kU/L (range)	7.8 (0.0–100.0)	7.2 (0.6–100.0)	p* = 0.11

*Note*: The data were expressed as median and range. A Mann–Whitney test (p*) and Chi^2^ Pearson's test (*p*) were performed in order to compare independent samples.

Abbreviation: VIT, venom immunotherapy.

^a^ II‐degree systemic reactions were significantly more frequent in the group of older patients (*p* < 0.05).

^b^ IV‐degree systemic reactions were significantly more frequent in the group of young patients (*p* < 0.05).

^c^ Significantly less frequently in the group of young patients than in the group of older patients (*p* < 0.05).

The methodology of the BAT assessment and the entire study protocol were described and consistent with the published first part of the study.^7^

The effectiveness of VIT was evaluated using BAT, revealing a statistically significant decrease in CD63 reactivity in the mean of about 86%–88% from the base for older patients similarly, as in young 84%–85% (*p* > 0.05) after 6 months of VIT. Similar trends were observed after next 6 months, and a comparable decrease in basophil activity was maintained at the level after 12 months. The results are presented in Figure 1A–C. After analyzing the BAT results after 6 and 12 months, no significant differences were found between age groups or vaccine types. In the entire study group of patients, 4.9% had negative (regardless of the interview and positive tests and IgE against bees) BAT results, while 3.6% showed no notable improvement after VIT compared to baseline. The obtained BAT values are presented for exposure to 1 μg/mL, as previous data indicate that this criterion provides the optimal balance of sensitivity and specificity when using standardized calibration curves.^7^

**FIGURE 1:**
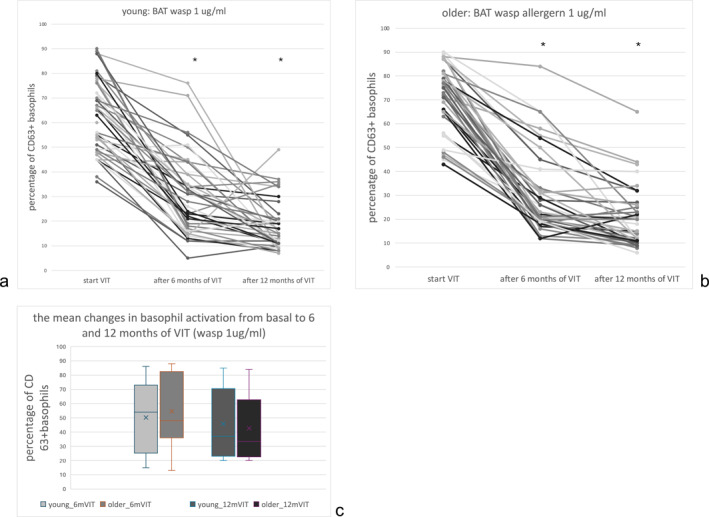
BAT in the groups of young (A) and older patients (B) at the start and after 6 and 12 months of VIT, and comparison of mean changes in CD63+ basophil activity between study groups (C). BAT, basophil activation test was performed with different concentrations of wasp venom extract; VIT, immunotherapy to wasp venom; however, only data for 1.0 μg/mL were presented for each group, as changes in two points: percentage of basophiles expressing CD63+ and significant depression after 6 months of VIT and comparable after 12 months of VIT in most patients in each group (*p < 0.05).

A few observations have been made regarding monitoring of VIT effectiveness using BAT, but not in the older homogeneous group. Most authors present BAT as a helpful method for the final qualification for VIT.^4^, ^5^, ^6^, ^8^ A significant decrease in the activity of stimulated basophils after starting immunotherapy and the maintenance of this trend after a year of treatment is consistent with the observations of other authors.^8^, ^9^

Previous observations have shown a decrease of basophil activation using mostly submaximal concentrations of insect venom extracts in study up to 18 months after the start of VIT. In contrast, a lower basophil reactivity was found in these studies after 2 years of treatment.^8^, ^9^

Therefore, this present study will continue to obtain long‐term results, particularly in older patients, to order to assess the long‐term effectiveness of VIT in this group.

## AUTHOR CONTRIBUTIONS

**Andrzej Bozek**: Conceptualization; methodology; software; data curation; supervision; formal analysis; writing—review and editing; investigation. **Martyna Miodonska**: Validation; methodology; investigation; software; supervision; data curation. **Aleksandra Mitka**: Investigation; validation; visualization; project administration; formal analysis; supervision; data curation. **Dominika Sadowska**: Investigation; funding acquisition; writing—original draft; writing—review and editing; validation; formal analysis. **Janne Winterstein**: Conceptualization; validation; supervision; resources; data curation; writing—review and editing; funding acquisition. **Radosław Gawlik**: Supervision; data curation; software; validation; investigation. **Marita Nittner‐Marszalska**: Conceptualization; investigation; funding acquisition; validation; methodology; writing—review and editing; project administration; resources; supervision.

## CONFLICT OF INTEREST STATEMENT

The authors declare no conflicts of interest.

## Data Availability

The data that support the findings of this study are available on request from the corresponding author. The data are not publicly available due to privacy or ethical restrictions.

## References

[1] Alvaro‐Lozano M , Akdis CA , Akdis M , et al. EAACI allergen immunotherapy user's guide. Pediatr Allergy Immunol. 2020;Suppl 25:1‐101. 10.1111/pai.13189 PMC7317851 32436290

[2] Roberts G , Pfaar O , Akdis CA , et al. EAACI guidelines on allergen immunotherapy: allergic rhinoconjunctivitis. Allergy. 2018;73(4):765‐798. 10.1111/all.13317 28940458

[3] Dhami S , Zaman H , Varga EM , et al. Allergen immunotherapy for insect venom allergy: a systematic review and meta‐analysis. Allergy. 2017;72(3):342‐365. 10.1111/all.13077 28120424

[4] Eberlein B , Brockow K , Darsow U , Biedermann T , Blank S . Basophil activation test in Hymenoptera venom allergy. Allergol Select. 2024;19(1):293‐298. 10.5414/alx02522e PMC11361271 39211355

[5] Kucera P , Cvackova M , Hulikova K , Juzova O , Pachl J . Basophil activation can predict clinical sensitivity in patients after venom immunotherapy. J Investig Allergol Clin Immunol. 2010;20:110‐116. 20461965

[6] Ruëff F , Bauer A , Becker S , et al. Diagnosis and treatment of Hymenoptera venom allergy. Allergol Select. 2023;2(7):154‐190. 10.5414/ALX02430E PMC10580978 37854067

[7] Bożek A , Winterstein J , Pawłowicz R , et al. Safety and efficacy of VIT against wasp venom in ultra‐rush protocols in patients older than 60 years. Vaccines (Basel). 2024;12(5):547. 10.3390/vaccines12050547 38793798 PMC11125965

[8] Eržen R , Košnik M , Silar M , Korošec P . Basophil response and the induction of a tolerance in venom immunotherapy: a long‐term sting challenge study. Allergy. 2012;67(6):822‐830. 10.1111/j.1398-9995.2012.02817.x 22469017

[9] Rodríguez Trabado A , Cámara Hijón C , Ramos Cantariño A , Romero‐Chala S , García‐Trujillo JA , Fernández Pereira LMM . Short‐intermediate‐and long‐term changes in basophil reactivity induced by venom. Allergy Asthma Immunol Res. 2016;8(5):412‐420. 10.4168/aair.2016.8.5.412 27334779 PMC4921695

